# Munch and Move: evaluation of a preschool healthy eating and movement skill program

**DOI:** 10.1186/1479-5868-7-80

**Published:** 2010-11-03

**Authors:** Louise L Hardy, Lesley King, Bridget Kelly, Louise Farrell, Sarah Howlett

**Affiliations:** 1 Prevention Research Collaboration, School of Public Health, University of Sydney, NSW Australia

## Abstract

**Background:**

Early childhood services have been identified as a key setting for promoting healthy eating and physical activity as a means of preventing overweight and obesity. However, there is limited evidence on effective nutrition and physical activity programs in this setting. The purpose of this study was to evaluate *Munch and Move*, a low-intensity, state-wide, professional development program designed to support early childhood professionals to promote healthy eating and physical activity among children in their care.

**Methods:**

The evaluation involved 15 intervention and 14 control preschools (n = 430; mean age 4.4 years) in Sydney, New South Wales, Australia and was based on a randomised-control design with pre and post evaluation of children's lunchbox contents, fundamental movement skills (FMS), preschool policies and practices and staff attitudes, knowledge and confidence related to physical activity, healthy eating and recreational screen time.

**Results:**

At follow up, FMS scores for locomotor, object control and total FMS score significantly improved by 3.4, 2.1 and 5.5 points more (respectively) in the intervention group compared with the control group (P < 0.001) and the number of FMS sessions per week increased by 1.5 (P = 0.05). The lunchbox audit showed that children in the intervention group significantly reduced sweetened drinks by 0.13 serves (i.e., 46 ml) (P = 0.05).

**Conclusion:**

The findings suggest that a low intensity preschool healthy weight intervention program can improve certain weight related behaviours. The findings also suggest that change to food policies are difficult to initiate mid-year and potentially a longer implementation period may be required to determine the efficacy of food policies to influence the contents of preschoolers lunchboxes.

## Introduction

Obesity prevention during early childhood is a priority in Australia and internationally, with approximately 15% of four year old Australian children classified as overweight and 6% obese [1]. Many of the behaviours linked to unhealthy weight gain, such as eating habits, food preferences, sedentary behaviours and enjoyment of physical activity are formed during the early period of life before commencing school [2,3], indicating that this age group are an important target population for the prevention of lifestyle habits associated with the development of overweight and obesity.

In Australia, more than 60% of four year old children attend preschool [4]; and thus preschools and other early childhood education and care (ECEC) services (e.g., long day care centres, family day care) have been identified as a key setting for promoting healthy eating and physical activity to children [5-7]. Through the *Children's Services Regulation 2004*, early child care services in NSW must develop and maintain a food and nutrition policy that are consistent with the Dietary Guide for Children [8]. For ECEC services such as preschools, which do not provide children with food, it is parents who provide the food which their child takes to preschool and the extent of food rules and reinforcement of those rules by preschools for food brought from home is not known. There are no definitive guidelines for ECEC services in regards to children's physical activity and non-educative exposure to screens (i.e., computers and television).

There is good evidence to support the need to develop interventions which target weight-related behaviors in preschool aged children. National data indicate that energy dense nutrient poor foods (i.e., 'extra' foods) contributed to approximately one-third of preschool aged children's energy intake [9] and that 'extra' foods displace core foods, which maybe compromising the nutritional status of very young children [10]. Furthermore, many 2-3 year olds are not meeting a number of core nutritional requirements [11]. National guidelines recommend that preschoolers should have limited exposure to television and videos/DVDs [12]; however according to the Longitudinal Study of Australian Children, 90% of four-five year olds spend more than two hours per day watching television, videos/DVDs [13]. The measurement of physical activity in very young children is problematic so information on preschoolers' physical activity levels is limited. One Australian study of preschool aged children showed that just over half (56%) spent three or more hours a day [14] in 'active play' on weekdays and just under 80% on weekends [15]. A key correlate of children's physical activity is the acquisition of fundamental movement skills [16] yet the evidence shows that mastery of these skills are low when children enter school [17].

Despite the documented high prevalence of overweight and obesity among preschool aged children [1,18], recent reviews indicate that only a few healthy weight interventions have been conducted and evaluated in the early childhood setting [5,19,20]. The paucity and different study methodologies has led to mixed results on changes in adiposity and weight related behaviors, indicating further intervention work is required to identify successful strategies that are feasible for widespread implementation.

In New South Wales (NSW) Australia, this program gap was addressed through the development of a professional development program for early childhood educators and carers called *Munch and Move*. Munch and Move was funded by the NSW Department of Health and designed for large-scale implementation across NSW, initially in 900 preschools; to be followed by a second implementation phase across 2,000 long day care centers. This paper describes the Munch and Move intervention program in detail and the evaluation of the effectiveness of the program in a small sample of NSW preschools.

## Methods

### Description of Munch and Move

Munch and Move was a key initiative of the *NSW Government's Plan Preventing Overweight and Obesity in Children, Young People and their Families 2009-2011*. Munch and Move was developed as a professional development program for early childhood workers, specifically to assist preschools and long day care centres promote strategies within their centres that encourage children's healthy eating, active play, and fundamental movement skills. The program aimed to increase the skills and confidence of staff working in preschools and long day care centres across NSW, as well as influence the policies and practices in the setting. Five key messages formed the basis of the Munch and Move program;

• Choose water as a drink;

• Eat fewer snacks and select healthier snack alternatives;

• Eat more fruit and vegetables;

• Get active for an hour or more each day; and

• Turn off the television and computer and get active.

The state-wide implementation of the Munch and Move involves collaboration with Area Health Services and the early childhood sector. Further, because of the large scale implementation of the program across all preschools and long day care centres in NSW, it was specifically designed as a low intensity and sustainable program, focusing only on professional development of staff and not on other factors which influence weight related behaviours of preschool children.

The components and implementation model of the *Munch and Move *program were based on formative research drawn from focus group work [6], survey findings [17] and were influenced by (i) extensive consultation with key stakeholders from the early childhood sector including the NSW Early Childhood Healthy Eating and Physical Activity Working Group, Kindergarten Union, Childcare NSW and ECTARC (an early childhood training organisation); (ii) consultation with Area Health Services; (iii) the policy and regulatory environment in which preschools and long day care centres operate within NSW; (iv) components and structure of 'Tooty Fruity Vegie/Fun Moves' (an intensive preschool based program implemented in Northern NSW [21,22]) and 'Lets Get Active' (Queensland Department of Sport and Recreation) and; (v) activities contained in Caring for Children manual [8]. This process was to ensure the program was relevant and appropriate to the early childhood sector and to develop strategies to influence systems and build capacity within the early childhood sector so as to influence healthy eating, increase games based active play and fundamental movement skills, and decrease recreational screen time in children 3-5 years of age.

The three core components of Munch and Move program were to provide: (i) a one day professional development workshop for preschool staff, delivered by a specialized early childhood training organization.; (ii) resources for preschools which included a manual and a small grant to support staff to attend training or purchase physical activity equipment for the preschool and; (iii) contact with health promotion professionals from local Area Health Services, to provide additional advice to preschools to support the delivery of the program. (For the evaluation study a dedicated project officer supported the program delivery.)

Briefly, preschool and long day care centres nominated a staff member to attend the one day workshop which provided training on (i) healthy eating and ways of incorporating food-based activities into their education program; (ii) physical activity and ways of incorporating fun, games-based skills activities into their program; (iii) strategies to encourage children to limit their recreational screen time; (iv) providing opportunities for children to engage in unstructured physically active play and; (v) developing and implementing healthy nutrition and physical activity fundraising policies within their setting.

The programs' manual was developed in collaboration with the training organisation (ECTARC), NSW Early Childhood Physical Activity and Healthy Eating Working Group and health promotion officers from the NSW North Coast Area Health Service. The manual contained removable, laminated pages with a range of games and learning experiences related to healthy eating, and fundamental movement skills (FMS) activities designed to develop locomotor, object control and stability skills. Additionally, examples of a physical activity, screen time and nutrition policy statements were included which could be adapted or adopted by the preschool. Other Munch and Move resources included a lanyard with a series of cards attached that contained pictures and text of the performance criteria for each FMS; fact sheets for noticeboards, Munch and Move poster and 'snake and ladders' game which were based on the five key Munch and Move messages.

### Evaluation Design Methods and Participants

The evaluation was conducted on a small set of NSW preschools, and conducted in parallel with the state-wide implementation of the program. The evaluation of Munch and Move program was based on a cluster randomization design, with outcome measures taken pre and post intervention. All preschools operating under the auspices of the NSW Department of Education and Training located in the Sydney (n = 15), Western Sydney (n = 22) and South Western Sydney (n = 24) education regions of NSW were invited to participate in the study (n = 61). In order to detect a difference in outcome behaviour of 10% between the intervention and control groups with 80% power and significance level of 0.05, it was estimated that 177 children were required for each study arm.

Once a preschool consented to participate in the study, the parents of children were then invited to allow their child to participate in the study. Informed consent from children's parent or guardian was a requirement for participation. Ethics approval was granted by The University of Sydney Human Research Ethics Committee.

Data at both time points were collected by the same 10 researchers, who were trained on the data protocols and collected data in teams of 3-4 per preschool. Preschools were randomly allocated to the intervention or control group after baseline data were collected. Researchers collecting the data were blinded to the intervention allocation.

**Figure 1:**
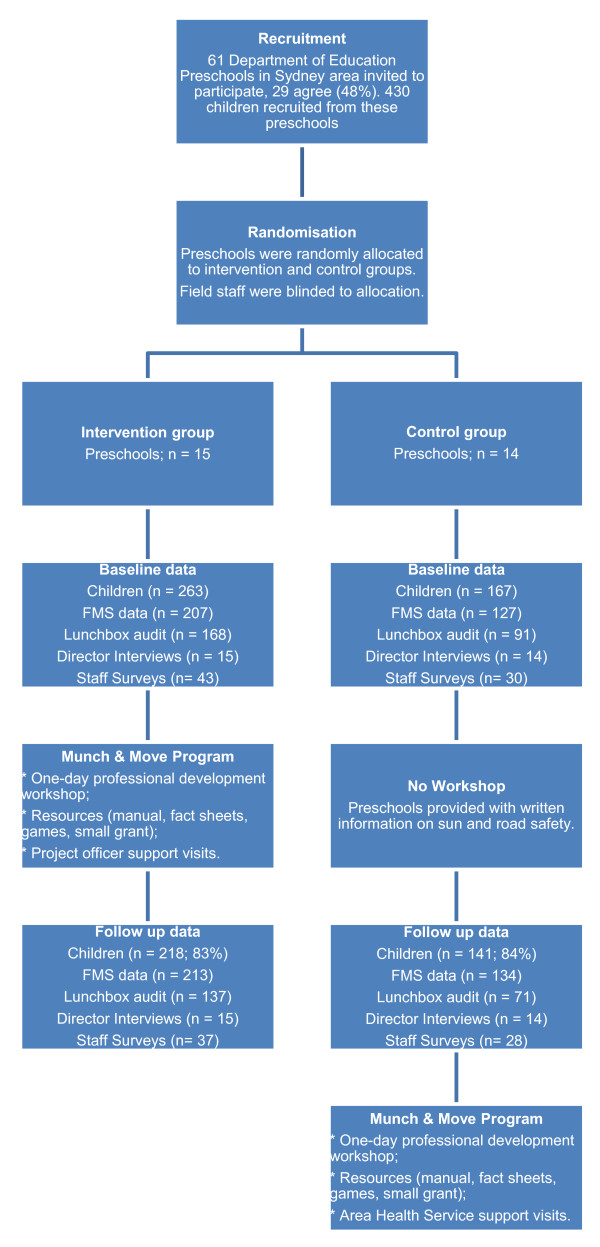
**Flow diagram of participants**.

Data were collected in May/June 2008 (pre-intervention) and November 2008 (post-intervention). Staff in intervention preschools attended a Munch and Move professional development workshop in late June 2008. Intervention preschools were visited on two occasions following the workshop, and provided with additional Munch and Move resources as required (e.g., FMS lanyards, snake and ladder games, posters). Control preschools received health information on unrelated topics (road safety and sun safety) during this period. Staff from the control preschools attended the workshop in late November 2008. A flow diagram of the evaluation is provided in Figure 1.

### Measurements

#### Demographic information

Parents completed a written survey which included information on their child's sex, date of birth, postcode of residence and the main language spoken at home. Postcode of residence was used as a proxy for socioeconomic status, based on the Australian Bureau of Statistics' Index of Relative Socioeconomic Disadvantage (IRSD) [23] and was used to rank children's socio-economic status (SES) (low or middle/high). Language spoken most at home was used to categorise students into English-speaking and non-English-speaking cultural backgrounds.

#### Lunchbox audit

Preschools were advised of the date of the visit for data collection 24 hours in advance and asked not to disclose this to parents as this may have affected the contents of the child's lunchbox. Lunch box audits were conducted in the preschool kitchen or private room, out of sight of the children, at the start of the preschool day, prior to the children accessing any of their food or beverages.

Details of all food and beverage lunchbox items, including brand name, product description, and weight were recorded. Electronic scales (Tanita Kitchen Scales KD160) were used to measure the weight of all food and beverages items. All food and beverages were classified as one of seven major food and beverage categories; fruit, vegetables, dairy, breads and cereals, healthy beverages (i.e. water, milk), 'extra' foods, or 'extra' drink, and also categorized according to 26 food and beverage sub-groups. Coding and classification of food items was conducted by a senior research dietitian (BK).

Food and beverage classification and serving size was based on the Australian Guide to Healthy Eating (AGHE) [24], where one serve of fruit was equivalent to 150 g; one serve of vegetables 75 g; one serve of extra foods 600 kJ; and 375-730 kJ represented one serve of dairy. Extra foods and drinks are those which dietary guidelines recommend should only be eaten occasionally (not every day), as they are higher in fat and/or sugar, energy or salt, and contain very few nutrients. 'Extra' foods included muesli bars, sweet biscuits, chips, confectionary, cakes, muffins, pastries, and high fat savory snacks. 'Extra' drinks included soft drinks, fruit juice and fruit juice drinks.

The overall contents of each lunchbox were also categorised by the extent to which they corresponded to nutritional recommendations on limiting extra foods and consuming core foods. Three lunchbox categories were developed:

(i) *balanced*: containing at least a sandwich or home-cooked meal and either fruit or vegetables, with the allowance of one extra serve (food or beverage);

(ii) *over-loaded with extra food or beverage*: containing greater than 1 extra serve (food or beverage), in addition to the contents of the balanced lunchbox; and

(iii) *unbalanced and/or over-loaded with extra food or beverage*: containing one or fewer balanced lunchbox components and/or too many extra serves (food or beverage) (> 1), or no lunchbox.

An upper threshold of one extra food item was calculated to represent a balanced lunchbox. School children are estimated to consume approximately one-third of their total energy intake at school [25]. As the AGHE recommends between one and two extra foods per day for preschool aged children, 0.7 serves of extra food were estimated to meet this recommendation. This value was rounded up to one serve for practical purposes.

#### Fundamental movement skills

Children were assessed on eight fundamental movement skills (FMS): four locomotor subtest skills (run, gallop, hop, horizontal jump) and four object control subtest skills (striking a stationary ball, catch, kick, overhand throw) using the process-oriented Test of Gross Movement Development (TMGD-2) checklist [26]. These skills were selected for assessment because they facilitate the development of more advanced movement skills. A more detailed description of the study assessment method is available elsewhere [27]. Briefly, each skill comprises three to five performance criteria which were scored as either present or absent, on two trials and summed to give a total score for locomotor skills (maximum score = 34) object control skills (maximum score = 32) and a total FMS score (maximum score = 66).

Prior to testing the field team were trained on the administration of the TGMD-2 by one of the authors (LH) who has experience in FMS assessment. The inter-rater reliability, determined by the intra-class correlation coefficient, was 0.9 for the total test. The children were tested in small groups (one assessor per child) in an outside area and each skill was demonstrated prior to testing, including providing a verbal description of the skill. The children were allowed to practice each skill before being scored on two test trials. If an assessor was unsure about a child's performance on a skill the child was asked to repeat the skill and the other assessors were consulted. The child was then scored according to agreement among the assessors. There was no specific order to administer the tests and a standard scoring sheet was used to record each child's performance.

#### Preschool policies and practices

The Director of each preschool was interviewed by one of the field team to ascertain preschool policies and practices related to physical activity, healthy eating, and the time which children spent watching television/DVDs (i.e. recreational screen time). Teachers and teachers-aids were also asked to complete a questionnaire regarding their attitude, knowledge, and confidence related to physical activity, healthy eating, and recreational screen time. At follow-up, the Directors and teachers were re-interviewed and surveyed using the same instruments and staff from intervention preschools who attended the Munch and Move workshop were asked to provide feedback on their experience of the program.

### Analysis

Data were analysed using SPSS Complex Samples (Version 16 for Windows, Chicago, IL, USA) to account for the clustered design of the study and adjust for the standard errors and 95% confidence intervals. The CSPlan procedure was used to allow for clustering within preschool class. The efficacy of Munch and Move was determined using generalized linear modelling (GLM) for continuous variables and logistic regression models for categorical variables, with the follow-up measure as the dependent variable, group as the independent variable, the baseline measure as the covariate with measures adjusted for sex, SES and English-speaking background. Statistical significance was set at P < 0.05.

## Results

### Participant characteristics

Sixty-one preschools were invited to participate in the study and 29 (48%) agreed to be involved. The number and proportion of preschools from each education region who participated were Sydney (n = 4; 27%), Sydney West (n = 12; 55%) and South Western Sydney (n = 13; 54%). Fifteen preschools were randomly allocated to the intervention group and 14 to the control group. Consent was obtained from the parents of 430 children within these schools (54% response rate; intervention group n = 263; control group n = 167). The demographic characteristics of the sample are provided in Table 1.

**Table 1:** Descriptive characteristics of preschools and children

	Base-line		Follow-up	
	**Intervention**	**Control**	**Intervention**	**Control**

Preschools (n)	15	14	15	14
*Preschool staffing (n)*				
Teachers	26	17	24	17
Teachers' aide	17	13	13	11
*Preschool staff experience (mean years)*				
Teachers	4.5	6.0	5.0	7.2
Teachers-aid	11.1	8.9	12.2	8.9
*Students (n)*	263	167	218	141
Boys (%)	49.4	50.3	47.6	46.3
*Age (yrs SD)*	4.4 (0.5)	4.5 (0.3)	4.9 (0.5)	4.9 (0.3)
*Days attending preschool (%)*				
2 days/week	22.4	11.0	20.0	10.6
3 days/week	21.3	42.3	20.6	38.2
4 days/week	7.9	4.3	8.2	5.7
5 days/week	48.4	42.3	48.2	44.7
*Socioeconomic status (%)*				
Low	47.5	44.3	47.7	46.3
Middle/high	52.5	55.7	52.3	53.7
*Cultural background (%)*				
English-speaking	57.6	40.7	37.0	50.4
Non-English speaking	42.4	59.3	63.0	49.6

At follow-up, 347 (80%) of children were available. Of those, 16% and 17% of children in the control and intervention groups, respectively, were lost to follow up, resulting in 218 children in the intervention group and 141 in the control group at follow-up. The primary reasons for loss to follow up were that the children were no longer at the preschool (n = 44) or were absent on the day of data collection (n = 22). There were no significant differences between the intervention and control group at follow up according to sex, age and SES; however the intervention group comprised a higher proportion of children from non-English speaking backgrounds (P = 0.03).

The pre-post findings of the mean FMS subtest scores, serves of selected food items in the children's lunchboxes and the differences between intervention and control children, adjusted for baseline values, sex, SES and English-speaking background are reported in Table 2.

**Table 2:** Regression coefficients for behavioural outcomes

Outcome	Baseline		Follow-up		Difference at follow up^1^	
	Intervention (n = 207)	Control (n = 127)	Intervention (n = 213)	Control (n = 134)	Adjusted I-C difference (95%CI)^2^	P value
***Fundamental movement skills (mean scores SD)***						
Locomotor score	23.1 (6.9)	21.3 (6.5)	25.2 (6.6)	22.1 (6.6)	3.41 (0.77, 6.05)	0.01
Object control score	20.0 (6.3)	19.0 (5.7)	22.8 (5.4)	20.7 (5.7)	2.07 (0.76, 3.41)	0.003
Total FMS score	43.3 (10.5)	40.5 (9.1)	48.0 (9.9)	42.8 (9.9)	5.33 (1.95, 8.71)	0.003
***Lunchbox items (mean serves SD)***	**(n = 168)**	**(n = 91)**	**(n = 137)**	**(n = 71)**		
Fruit serves	1.0 (0.9)	1.0 (0.8)	0.9 (1.0)	1.0 (0.9)	-0.05 (-0.36, 0.26)	0.75
Vegetables serves	0.03 (0.2)	0.12 (0.4)	0.14 (0.5)	0.15 (0.5)	0.12 (-0.05, 0.30)	0.16
Snack serves	1.35 (1.3)	0.83 (1.1)	1.40 (1.4)	0.94 (1.2)	0.06 (-0.34, 0.46)	0.75
Sweetened drink serves^3^	0.49 (0.7)	0.55 (0.6)	0.41 (0.5)	0.52 (0.5)	-0.13 (-0.27, 0.002)	0.05
Total extra food/drink serves	1.78 (1.5)	1.31 (1.3)	1.77 (1.5)	1.42 (1.4)	-0.6 (-0.45, 0.33)	0.76
*Lunch boxes containing > 1 serve of extra food/drink (%)*					**Odds ratio (95% CI)^4^**	**P value**
Extra foods > 1 serve (%)	49.4	29.7	51.4	37.5	0.90 (0.48, 1.70)	0.74
Extra drinks > 1 serve (%)	11.9	14.3	6.8	9.1	0.52 (0.12, 2.19)	0.53
Extra food/drink > 1 serve (%)	61.9	50.5	62.8	48.9	0.86 (0.43, 1.74)	0.67
*Lunchbox categories (%)*						
Balanced (%)	29.8	39.5	29.1	39.8	0.85 (0.32, 2.25)	0.72
Overloaded with extras (%)	39.2	34.1	34.5	36.4	0.74 (0.33, 1.65)	0.72
Unbalanced (%)	31.1	26.4	36.5	23.9	1.18 (0.44, 3.14)	0.72

^1 ^The models for the differences between intervention and control groups were adjusted for baseline values, sex, SES, English-speaking background and clustering of preschools; ^2^GLM; ^3^Sweetened drinks = 100% fruit juice, fruit juice, soft drinks and colored drinks; ^4^Logistic regression models.

### Fundamental movement skills

**Figure 2:**
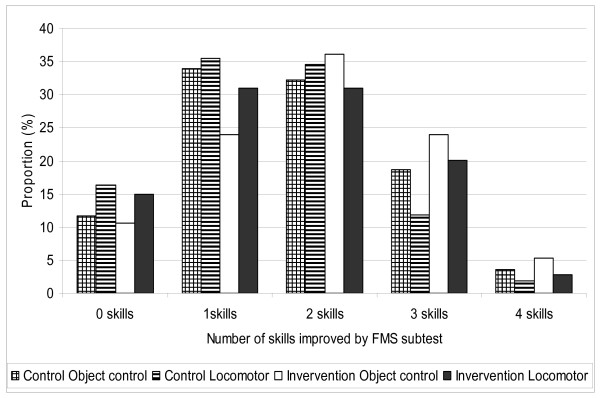
**Proportion (%) of children who demonstrated improvement in none, one, two, three or all four FMS by FMS subtests (locomotor or object control) and intervention group (control, intervention)**.

On the day of the preschool visit at both pre and post testing, not all children wanted to participate in the FMS assessment. FMS sub-tests scores for locomotor, object control and total FMS score significantly improved by an estimated 3.4, 2.1, and 5.5 points more (respectively) in the intervention group compared with the control group. The proportion of children in the intervention and control groups who demonstrated no improvement or an improvement in one or more locomotor and object control skills at follow-up are shown in Figure 2. Overall, children in the intervention group showed a larger, but non-significant, improvement across a range of skills compared with children in the control group. In the intervention group, 54% of children demonstrated improvement in 2 or more locomotor skills compared with 48% of children in the control group (χ^2 ^= 0.92; P = 0.34). Similarly a higher proportion of children in the intervention group improved on two or more object control skills compared with children in the control group (65% vs 55%, respectively; χ^2 ^= 3.56; P = 0.06).

### Lunchbox contents

Not all children were involved in the lunchbox audit. Four preschools operated on a half day basis, four preschools provided food for children and not all children were present on the day of the visit, resulting in 259 and 208 lunchboxes for analysis pre and post-intervention, respectively. In terms of the serves of food and drink items in children's lunchboxes, at follow-up the number of sweetened drink serves decreased by an estimated 0.13 serves (i.e., 46 ml) (p = 0.05) in the intervention group compared with control group.

There was no significant changes in serves of fruit (p = 0.75), snacks (p = 0.75) and total extra food/drink (p = 0.79) in the intervention group compared with control group. The number of vegetable serves increased by 0.12 serves (i.e., 9 grams) in the intervention group compared with control group, but this was not statistically significant. Similarly, there were no significant changes in the proportion of lunch boxes that contained 'extra' food or drink items, or in the three categories used to define the overall contents of the children's lunchboxes.

### Preschool policy and practices

Table 3 shows changes the preschools' physical activity practices. At baseline and follow-up, all preschools reported that unstructured active play time was scheduled every day. By comparison, daily structured active play time was scheduled at 48% and 62% of preschools at baseline and follow up respectively. Overall, there was no significant change in the frequency or time scheduled for structured and unstructured active play time in the intervention, compared with control preschools. While the time allocated to FMS activities increased in both groups between baseline and follow up, this was not statistically significant (p = 0.6); however the frequency of delivering FMS activities increased by an estimated 1.5 sessions per week in the intervention compared with control preschools (p = 0.05). In effect, at follow up the intervention schools allocated 75.52 minutes per week to FMS activities, and control schools 44.08 minuets per week.

**Table 3:** Regression coefficients for preschool physical activity environment^1^

Outcome	Baseline*		Follow-up*		Difference at follow up	
	Intervention	Control	Intervention	Control	Adjusted I-C difference (95%CI)	P value
*Physical activity*						
Structured play time (mins per session)	24.3	31.4	30.8	31.4	0.09 (-11.6, 11.8)	0.90
Frequency of structured play (sessions per/wk)	3.8	3.0	3.5	3.6	0.02 (-1.5, 1.5)	0.90
Unstructured play (mins per session)	77.0	77.1	73.0	65.4	7.7 (-15.6, 31.0)	0.50
Frequency of unstructured play (sessions per/wk)	5.0	5.0	5.0	4.8	-	-
FMS activities (mins per session)	5.3	15.0	23.6	23.2	3.4 (-9.7, 16.5)	0.60
Frequency of FMS activities (sessions per/wk)	1.3	1.9	3.2	1.9	1.5 (0.01, 2.9)	0.05

^1^The models for the differences between intervention and control groups were adjusted for baseline values, sex, SES, English-speaking background and clustering of preschools.

All preschools reported conducting some form of food based activities (e.g., cooking classes, taste tests, school garden etc) (data not shown) at baseline and follow-up; however there no significant changes in these activities in the intervention preschools compared with controls at follow up. At pre-intervention, the majority of Directors reported having rules concerning food and drink brought in from home. In some cases, the rules specified suggested foods, for example 'fruit for morning and afternoon tea', and in other cases non-perishable items, when no refrigeration facilities were available. Otherwise, food rules were mostly a statement of what not to bring to preschool, such as 'no chips or lollies', or a combined message, e.g., 'water, no juice until lunch, fruit for morning tea'.

There were no changes in preschool food policies at follow up and there were no apparent differences between control or intervention preschools. A few preschools included a comprehensive list of extra foods that were not allowed; some preschools excluded foods from their lists that are in fact appropriate for the preschool setting. It would appear that many preschool Directors found it difficult to know exactly which foods they should and should not include in lists of appropriate items. Additionally, Directors reported difficulties in changing food and drink policies and rules mid year, following the workshop.

Directors reported a variety of methods to communicate food rules and policies to parents, including letters to parents, speaking directly to parents, newsletters, notice boards, and orientation kits. Some Directors indicated that excluded foods were sent back home at the end of the day, or did so in combination with a note, e.g., 'send it home with slip saying "not healthy for children, please do not send"'. Others reported talking directly to parents about appropriate foods, as a means of implementing food rules and policies within the preschool.

### Staff survey

Table 4 shows the findings from surveys conducted with teachers and teachers-aids at the participating preschools (this includes staff who did not personally participate in the training) on their attitudes, confidence, and knowledge relating to physical activity, healthy eating, and small screen recreation. At follow up, there were small non significant changes in attitudes and confidence among both groups, but more so among the intervention staff compared with the control preschool staff. Similarly there no significant differences between the groups at follow up for knowledge about recommended guidelines for fruit vegetables and screen time. Knowledge regarding the correct serves of fruit and screen time in fact decreased among intervention staff.

**Table 4:** Prevalence of teacher and teachers-aid response to the attitudes, confidence, and knowledge survey (%)

	Pre-intervention		Post-intervention		
	Intervention (n = 43)	Control (n = 30)	Intervention (n = 37)	Control (n = 28)	P-value
**Attitudes (agreement with statement)**					
Teachers do not need to act as role models for being active	5.0	11.5	0.0	7.5	0.09
It is not the role of the teacher to teach movement skills	14.3	10.0	5.7	19.2	0.10
It is not important that children participate in structured active play	9.8	13.3	0.0	0.0	-
Safety concerns limit active play opportunities in the preschool setting	60.0	70.0	38.9	58.3	0.14
It is not the role of the teacher to teach about healthy eating	14.5	3.5	2.8	15.5	0.07
Parents should be able to send any type of food to school with their child.	7.3	3.3	0.0	3.7	0.25
It is alright to sell chocolates and sweets for fundraising	56.1	56.5	59.0	48.0	0.39
**Confidence**					
I am confident that I can teach movement skills to children.	97.6	90.0	97.3	95.7	0.73
I am confident in talking to parents about their child's movement skills.	80.5	96.7	97.0	90.9	0.33
I am confident in talking to parents about their child's lunchbox contents	76.9	89.7	89.7	95.8	0.40
Confident talking to parents about their child's television viewing	47.5	78.6	68.8	75.0	0.68
**Knowledge of recommended guidelines**					
Daily serves of fruit	100.0	93.0	94.5	96.0	0.79
Daily serves of vegetables	89.7	96.6	94.3	87.5	0.36
Recreational screen time (TV/DVDs) (hrs/day)	84.2	62.1	73.5	87.0	0.22

### Munch and Move workshop evaluation

One to two teachers from each preschool in the intervention group attended a Munch and Move workshop and completed an evaluation of the workshop (Table 5). Overall, all teachers rated the workshop as very helpful and the associated resources as useful or very useful. For the majority (75%), Munch and Move was the first professional development program they had attended in the past 5-years which focused on teaching active play, food, and health. Similarly, the majority of these teachers reported that their knowledge and confidence regarding the teaching and communication to parents of physical activity, healthy eating and screen time had improved as a direct result of the workshop.

**Table 5:** Munch and Move workshop participants' responses on previous training, knowledge and confidence gained from the workshop and anticipated change (n = 28)

Evaluation item	Response (n)	
**Prior professional development (PD)**	**Yes**	**No**
PD in past 5 years on teaching active play	8	20
PD in past 5 years on teaching about food and health	6	22
PD in past 5 years on communicating with parents	10	18
**Potential change in teaching practices (n = 27)**		
Anticipates changing food-based experiences in preschool	23	4
Anticipates changing active play experiences in preschool	26	1
**Knowledge & confidence**	**Increased**	**No change**
Knowledge of healthy eating in preschoolers	22	6
Knowledge of active play in preschoolers	22	6
Knowledge of FMS in preschoolers	28	0
Knowledge of how screen time can be limited	16	12
Confidence in delivering food-based activities	20	7
Confidence in teaching fundamental movement skills	27	1
Confidence in delivering structured active-play activities	25	3
Confidence in limiting time children spend on the computer	14	14
Confidence in talking to parents about healthy eating	19	9
Confidence in talking to parents about movement skills	21	7
Confidence in talking to parents about limiting screen time	20	8

## Discussion

The findings from this study suggest that the Munch and Move professional development program lead to an improvement in children's FMS and a decrease in the number of serves of sweetened drinks in children's lunchboxes. Furthermore, teachers in the intervention arm reported that Munch and Move was highly acceptable and appropriate for implementation in preschools with a mix of children from different socio-economic and cultural backgrounds. While there were no major significant changes in policies relating to physical activity and food, or in teacher's attitudes, confidence and knowledge relating to physical activity, healthy eating and recreational screen time, this may reflect the low-intensity of the program or potentially be attributed to the short implementation period in this evaluation study. While a longer implementation period prior to evaluation was required to better determine the efficacy and sustainability of the Munch and Move program this, unfortunately, was not feasible in this implementation trial.

An important finding was the limited extent of professional development on nutrition and physical activity that preschool teachers reported during the last five years. The teachers' positive evaluation of the Munch and Move workshop and resources suggests that early childhood staff would benefit from additional support and resources in teaching about weight-related behaviours, which is consistent with other research findings [6,28].

Another important finding was the significant increase in children's FMS scores in the intervention preschools. The acquisition of FMS which prepare children to engage in a wide range of physical activities, are not acquired naturally. Rather FMS require explicit teaching in order to develop proficiency or mastery of the skill [29]; and there is some evidence that early childhood staff may have limited knowledge about the individual components (i.e., performance criteria) of movement skills [30,31]. Teachers reported that information and resources on physical activity and the FMS activities in particular, within the '*Move' *component of the workshop, were highly relevant to their day-to-day teaching. The programs' FMS resources included a lanyard with a series of cards attached that contained pictures and text of the performance criteria for each skill on separate cards and a manual which contained removable, laminated pages with a range of games and learning experiences designed to develop locomotor, object control and stability skills.

Additionally, an example of a physical activity policy statement was also included which could be adapted or adopted by the preschool. Descriptive information on FMS proficiency among preschool aged children is limited [27], however our findings are supported by other studies which have also shown that targeted FMS programs increase proficiency in preschoolers and early primary school children [30,32-37].

The contents of children's lunchboxes remained unchanged following the intervention, with the exception of a decrease in sweetened drinks among the intervention preschooler's lunchboxes. Although information about sweetened drinks was a key learning outcome of the workshops, a 'drink water' social marketing campaign had been running concurrently in NSW around the time of the intervention, which may have had an external influence on the Munch and Move findings. A number of preschools however reported that it was difficult to introduce new food policies and rules halfway through the year, and so did not fully implement this part of the program.

The findings regarding the high frequency and excessive volume of extra foods in children's lunchboxes are consistent with results of studies on school children's lunchboxes [38]. This and other studies emphasise the need to improve the quality of young children's diets generally and the nutritional quality of their lunchboxes specifically. The extent of extra foods and consistency of patterns suggests that a much more comprehensive set of interventions may be required to ensure that parents are more aware of what food and drink items and portion sizes are 'extra' to children's nutritional requirements.

The evaluation of the Munch and Move intervention program had a number of limitations which suggest the results should be viewed as indicative and interpreted cautiously. Firstly, the period between baseline and follow-up measures was only 20 weeks. The preschools in this study did not have the opportunity to conduct a full year of implementation, which is particularly desirable for establishing food rules and activity routines. Secondly, the evaluation involved relatively small sample size, particularly the number of preschools and it is not known to what extent this sample of government preschools was representative of other preschools in NSW, which are managed by a range of community, non-government, and private organizations. Additionally, a number of children's lunchboxes were excluded from the audit due to unforeseen reasons (i.e., preschool was half day or provided food/beverages, or child was absent on the day) which resulted in smaller numbers than anticipated and may explain the null effect for lunchbox contents. As a check, a post-hoc test was conducted and indicated there was insufficient power to detect any significant difference based on the number of children who did participate at follow up. Importantly, however, this set of preschools represented a socio-economic mix of families and children, with particularly high proportions of disadvantaged and culturally diverse groups, who comprise important target groups for such programs.

*Munch and Move *was designed as a low intensity intervention and was efficacious to improve children's fundamental movement skills, but was not sufficient to produce changes in children's lunchboxes. Potentially the intervention may be more effective in producing changes in children's lunchbox contents if it were implemented by a preschool over a full year, with a strong initial focus on food policies and rules. A more intensive intervention to develop and support the work of early childhood staff may also add to the effectiveness of the program. In their review, Campbell and Hesketh [5] recognized that there was a lack of evidence regarding the effectiveness of low intensity interventions and that high intensity interventions often only produced small changes. There is scope to continue to develop and enhance the program.

The approach adopted by Munch and Move and similar programs has the potential to influence large numbers of children at low cost, and further evaluation of different versions with variations in intensity are warranted. Similarly, it is recognized that to reach a larger proportion of children and families, the implementation of the Munch and Move program would need to be extended to other early childhood services, such as family day centres.

## Competing interests

The authors declare that they have no competing interests.

## Authors' contributions

LH, LK, BK, LF and SH all contributed to the development of research questions and the design of the evaluation study, and the Ethics Committee submission. BK, LF and SH undertook data coding and data entry; LH, and BK were involved in checking the coding and analysis. All authors contributed to the interpretation of data and the writing of the manuscript. All authors read and approved the final manuscript.
